# Colloidal synthesis of ultrathin KFeS_2_ and RbFeS_2_ magnetic nanowires with non-van der Waals 1D structures

**DOI:** 10.1039/d5sc04592d

**Published:** 2025-09-12

**Authors:** Zhaohong Sun, Ngoc Pham, Shahab Derakhshan, Richard L. Brutchey

**Affiliations:** a Department of Chemistry, University of Southern California Los Angeles California 90089 USA brutchey@usc.edu; b Department of Chemistry and Biochemistry, California State University Long Beach California 90840 USA shahab.derakhshan@csulb.edu

## Abstract

Design of one-dimensional (1D) nanomaterials based on non-van der Waals (non-vdW) 1D chain structures is emerging as a new materials frontier, owing to their strong intrinsic anisotropy and broad compositional diversity. However, achieving ultrathin 1D morphology in such systems remains a significant challenge. In this work, we report the colloidal synthesis of ultrathin KFeS_2_ and RbFeS_2_ nanowires—representing the first fabrication of ultrathin 1D nanomaterials driven by non-vdW 1D crystal structures. The nanowires exhibit diameters of ∼5 nm and lengths of microns, with anisotropic growth directed by covalent [FeS_2_]^−^ chains. Magnetic characterization reveals significantly reduced antiferromagnetic transition temperatures and suppressed interchain ferromagnetic interactions, demonstrating pronounced size and morphology effects. Control experiments on structurally related materials indicate that direct nucleation of the 1D phase is essential for achieving the nanowire morphology. These findings establish a new synthetic pathway to an understudied family of non-vdW 1D nanomaterials, enabling exploration of their emergent quantum and magnetic properties.

## Abstract 2

Colloidal soft chemistry yields ultrathin KFeS_2_ and RbFeS_2_ nanowires with size and morphology-dependent magnetism, representing the first fabrication of ultrathin 1D nanomaterials driven by non-van der Waals 1D crystal structures.

## Graphical abstract

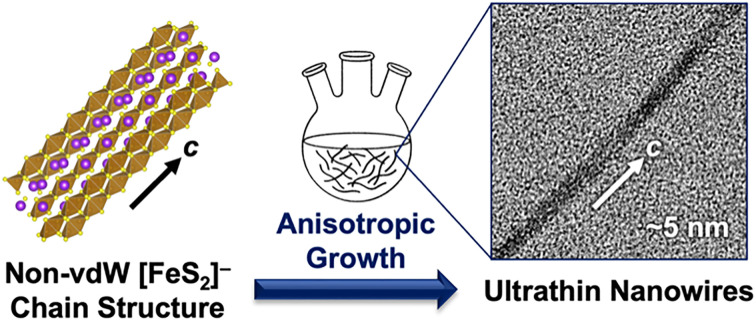

## Introduction

Low-dimensional materials—those with confined geometries in one or more spatial directions—have garnered interest due to their unique properties and versatile applications across photonics, sensors, magnetism, and quantum devices.^1–4^ In particular, the ability to isolate and manipulate two-dimensional (2D) materials has led to a surge of discoveries, beginning with graphene and rapidly expanding to a variety of layered van der Waals (vdW) compounds, such as transition metal dichalcogenides, black phosphorus, and hexagonal boron nitride.^5–7^ These materials benefit from weak interlayer vdW forces, which make exfoliation and solution-phase synthesis relatively straightforward, enabling in-depth exploration of their structure–property relationships.

In contrast, the structure-driven synthesis of one-dimensional (1D) materials remains considerably more challenging. While nanowires or nanoribbons have been synthesized from 1D or quasi-1D vdW structures such as transition metal trichalcogenides (*e.g.*, TiS_3_, TaSe_3_, and ZrTe_3_),^8–12^ only a few examples outside this family exist, such as Nb_2_Se_9_ and Bi_4_X_4_ (X = Br, I),^13,14^ due to the scarcity of coordination motifs that can maintain bonding anisotropy along a single axis.^15^ On the other hand, researchers have explored 1D nanomaterials derived from 3D non-vdW structures, such as metal, metal chalcogenides, and perovskites.^16–21^ These systems rely on a combination of weak crystallographic anisotropy, facet-selective ligand binding, and kinetic control to favor growth along one axis, thus making the synthetic strategies difficult to generalize.

**Fig. 1:**
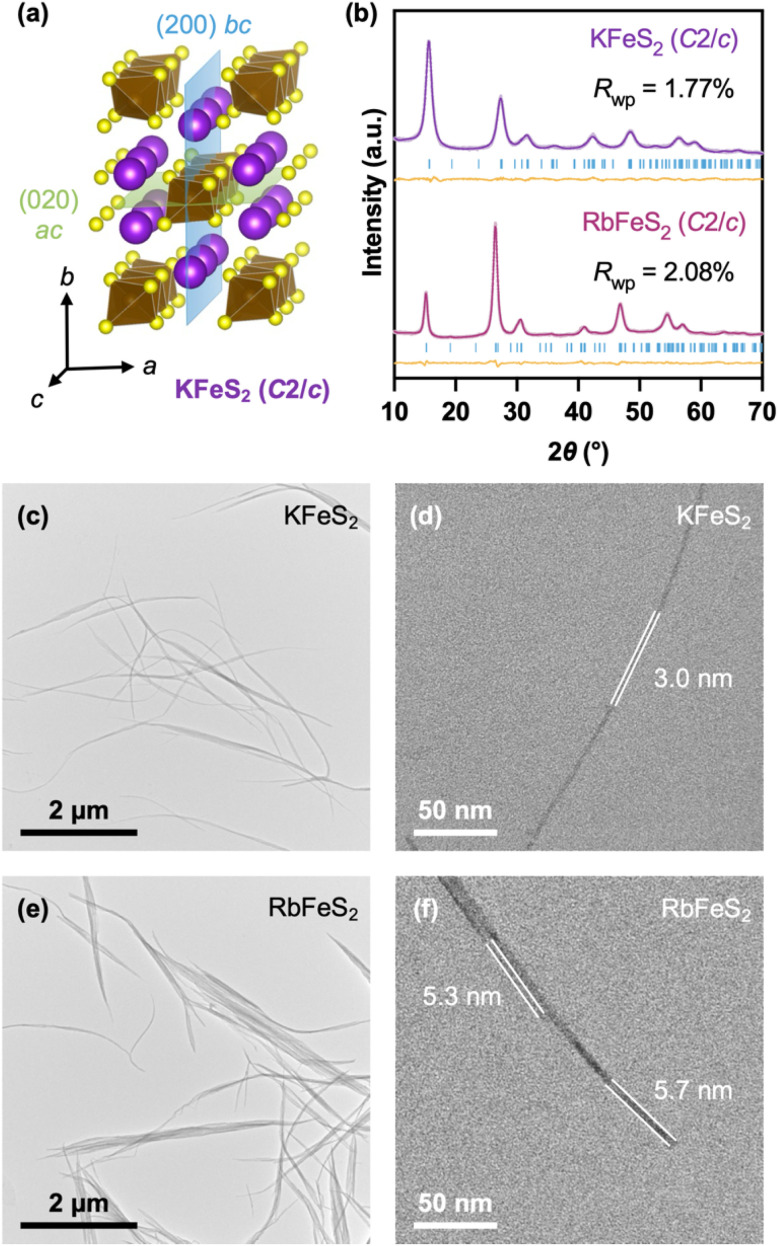
(a) Crystal structure of monoclinic KFeS_2_ (space group *C*2/*c*), featuring covalent [FeS_2_]^−^ chains along the *c*-axis, hexagonally packed among K^+^. (b) Rietveld refinement of powder XRD patterns of KFeS_2_ and RbFeS_2_ nanowires; refined structural parameters are listed in Table S1. Structure .cif files for KFeS_2_ (ICSD-68383) and RbFeS_2_ (ICSD-202381) were obtained from the Inorganic Crystal Structure Database (ICSD). (c–f) TEM images of (c and d) KFeS_2_ and (e and f) RbFeS_2_ nanowires. Additional images are provided in Fig. S1a and b.

Bridging these two regimes is a class of non-vdW 1D structures that feature strong covalent bonding along one axis and ionic bonding in orthogonal directions.^22^ This intermediate level of anisotropy enables directional crystal growth while expanding the accessible chemical space beyond 1D vdW structures. A representative family includes alkali metal-based ternary chalcogenides such as KFeS_2_, where covalent [FeS_2_]^−^ chains are stabilized by K^+^ (Fig. 1a).^23^ KFeS_2_ exhibits long-range antiferromagnetic (AFM) ordering originating from intrachain Fe–Fe superexchange, along with weak interchain ferromagnetic (FM) interactions.^24^ Such anisotropic magnetic behavior makes KFeS_2_ an ideal model system for probing how 1D morphological confinement influences magnetic properties.

While efforts have aimed to transform these 1D structures into highly anisotropic nanomaterials, prior reports suffer from poor dimensional confinement and limitations in product separation. In bottom–up hydrothermal synthesis, KFeS_2_ has been grown into thick “nanowires” averaging ∼160 nm in diameter, demonstrating the power of non-vdW 1D crystal structures in directing anisotropic growth.^25^ However, these nanowires remain relatively thick, and the requirement of iron foam substrates impedes the isolation of free-standing nanomaterials. Top-down chemical exfoliation of bulk KFeS_2_ has yielded nanoribbons with dimensions of ∼20 nm × 200 nm × 20 μm.^24^ Yet, this method suffers from low yield (<3%) and poor morphological selectivity, producing micrometer-sized nanosheets as byproducts. In both cases, the intrinsic 1D anisotropy of KFeS_2_ is not fully translated into the final nanomaterial, and the challenges in post-synthetic separation hinder the investigation of their physical properties. In contrast, colloidal soft-chemistry methods are powerful in controlling nanomaterial size and morphology through rapid nucleation and surface ligand stabilization, often yielding monodispersed nanocrystals only a few nanometers in size.^26^ No solid substrate is required for crystal growth, allowing for the scalable production of free-standing nanomaterials. Colloidal synthesis has been extensively developed for 2D vdW materials—particularly transition metal dichalcogenides—enabling the growth of ultrathin nanosheets down to the monolayer limit.^27–30^ However, this method has yet to be extended to 1D non-vdW structures.

In this study, we report the colloidal synthesis of ultrathin KFeS_2_ and RbFeS_2_ nanowires, which exhibit pronounced size effects in magnetic behavior. Our findings highlight the capacity of non-vdW 1D structures to drive strongly anisotropic growth in colloidal synthesis, establishing a new soft-chemistry route to a large family of 1D nanomaterials.

## Results and discussion

KFeS_2_ and RbFeS_2_ nanowires were synthesized using a method adopted from our previous study on AInSe_2_ (A = K, Rb, Cs) nanocrystals.^31^ Potassium or rubidium carbonate, iron(iii) acetylacetonate, and dibenzyl disulfide were dissolved in oleic acid and oleylamine and degassed at 140 °C for 1 h, which transformed the alkali metal carbonates into reactive oleate precursors. Then the suspension was heated to 350 °C and maintained at that temperature for 2 h, followed by thermal quenching to room temperature and purification with a hexanes/ethanol mixture. Rietveld refinement of powder X-ray diffraction (XRD) patterns revealed that the nanowires are phase-pure and adopted the same monoclinic (*C*2/*c*) structure as reported in the bulk (Fig. 1b and Table S1), with negligible shifts in atomic coordinates. Broad diffraction peaks and suppressed intensities of some reflections indicated nanoscale dimensions with strong preferred orientations (Fig. 1b). Transmission electron microscopy (TEM) imaging revealed an ultrathin nanowire morphology (Fig. 1c–f, S1a and b). KFeS_2_ and RbFeS_2_ nanowires exhibited diameters of 5.4 ± 1.1 nm (*N* = 72) and 6.3 ± 1.2 nm (*N* = 82), respectively (Fig. S1c); both had lengths of several μm, yielding aspect ratios on the order of 10^2^–10^3^. This confirms their highly anisotropic one-dimensional (1D) character. With cross-sections comprising only a few unit cells, these nanowires were significantly thinner than previously reported hydrothermally synthesized analogues (∼160 nm in diameter).^25^ There was no morphological heterogeneity as observed in chemically exfoliated nanomaterials, which contained a mixture of nanoribbons and large nanosheets.^24^ The nanowires were mostly in bundles, likely due to strong coulombic attraction between K^+^ and [FeS_2_]^−^, while some isolated nanowire sections could be observed.

Elemental compositions of the nanowires were characterized using scanning electron microscopy coupled with energy dispersive X-ray spectroscopy (SEM-EDX, Table S2). The KFeS_2_ nanowires possessed a composition of K_1.2_FeS_1.8_, revealing a 20% supersaturation of K^+^. Such supersaturation has been previously observed in FeS_2_-based K^+^-ion batteries, where the stoichiometry of K_*x*_FeS_2_ could be varied between 0.0–2.0 upon charging and discharging without a structural reconstruction.^32^ In contrast, RbFeS_2_ nanowires revealed a composition of Rb_1.0_FeS_2.3_ without Rb^+^ supersaturation. The bulkier size of Rb^+^ (ionic radius *r* = 1.61 Å) compared to K^+^ (*r* = 1.51 Å) makes it more difficult to intercalate in the structure as interstitials, thereby preventing supersaturation.^33^ High-resolution X-ray photoelectron spectroscopy (XPS) of both KFeS_2_ and RbFeS_2_ nanowires revealed broad, asymmetric Fe 2p_3/2_ peaks at 708.7 eV and Fe 2p_1/2_ peaks at 721.8 eV (Fig. S2). These features are consistent with previously reported spectra of KFeS_2_ and indicative of [Fe^III^S_4_] bonding environments.^25,34–36^ The spectral similarity between KFeS_2_ and RbFeS_2_ further suggests that K^+^ and Rb^+^ exhibit minimal covalent interaction with the [FeS_2_]^−^ chains, highlighting the ionic feature of the non-vdW chain structure.

**Fig. 2:**
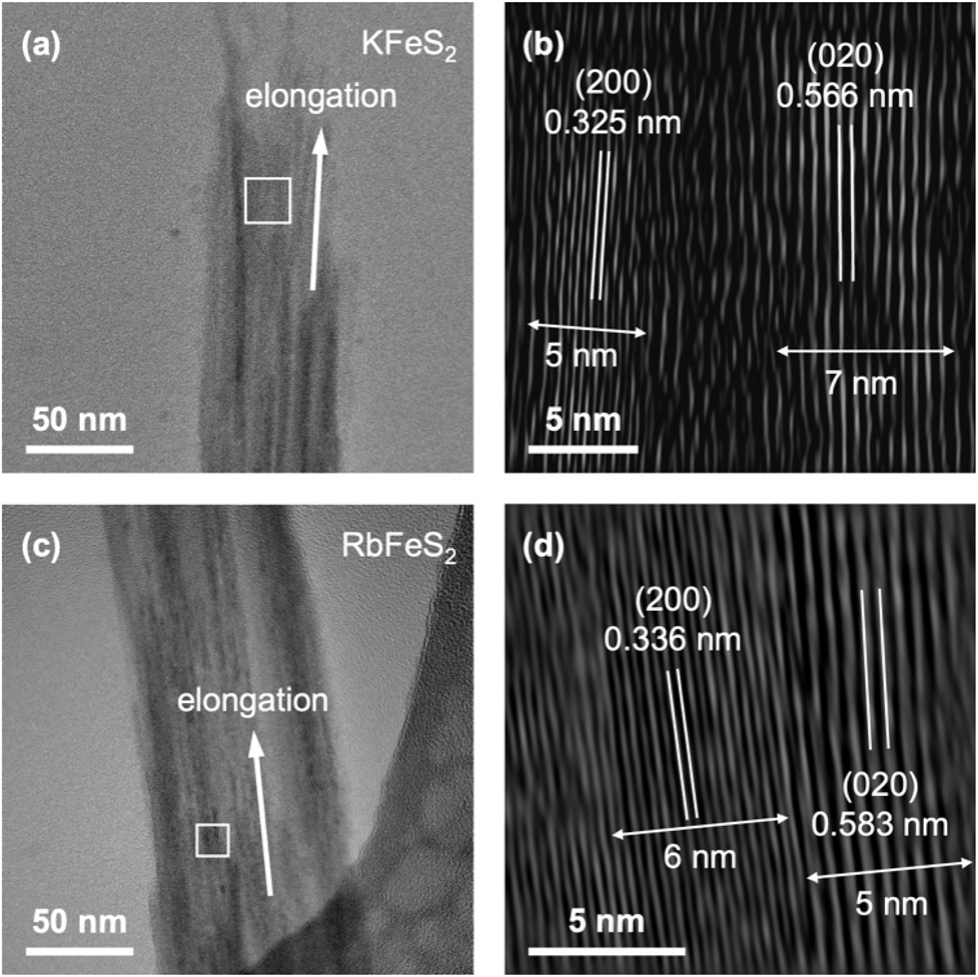
(a and c) TEM images of a bundle of (a) KFeS_2_ and (c) RbFeS_2_ nanowires. (b and d) FFT-filtered magnified sections of the white boxed regions in (a) and (c), respectively, showing ac and bc plane spacings parallel to the nanowire elongation direction. Unfiltered images are shown in Fig. S3a and b.

Given the anisotropic structure of KFeS_2_ and RbFeS_2_, the nanowires are likely growing along the crystallographic *c*-axis, parallel to the [FeS_2_]^−^ chains (Fig. 1a). To prove this, TEM images were collected at a magnification that allows for the simultaneous observation of macroscopic nanowire morphology and high-resolution crystal lattices (Fig. 2). Fig. 2a and c show bundles of KFeS_2_ and RbFeS_2_ nanowires along the vertical direction, respectively. Fast Fourier Transform (FFT) filtering of the white boxed areas yielded the high-resolution images shown in Fig. 2b and d, which revealed the (200) and (020) spacings on adjacent nanowires simultaneously, indicating that both *bc* and *ac* planes are parallel to the elongation direction. The nanowire growth thus must have occurred along the intersection of these two planes, *i.e.*, the crystallographic *c*-axis (Fig. 1a). This observation revealed that the non-vdW 1D structure defined by the covalent [FeS_2_]^−^ chains served as the driving force for the formation of the nanowire morphology. Meanwhile, measurements of the continuous areas with uniform lattice fringes revealed crystallographic domains 5–7 nm wide (Fig. 2b and d), corresponding to the thicknesses measured from isolated nanowire sections. When nanowires are well aligned at the eucentric height, the observed lattice fringes can extend over hundreds of nanometers (Fig. S3c–f), indicating long-range crystallinity along the *c*-direction.

**Fig. 3:**
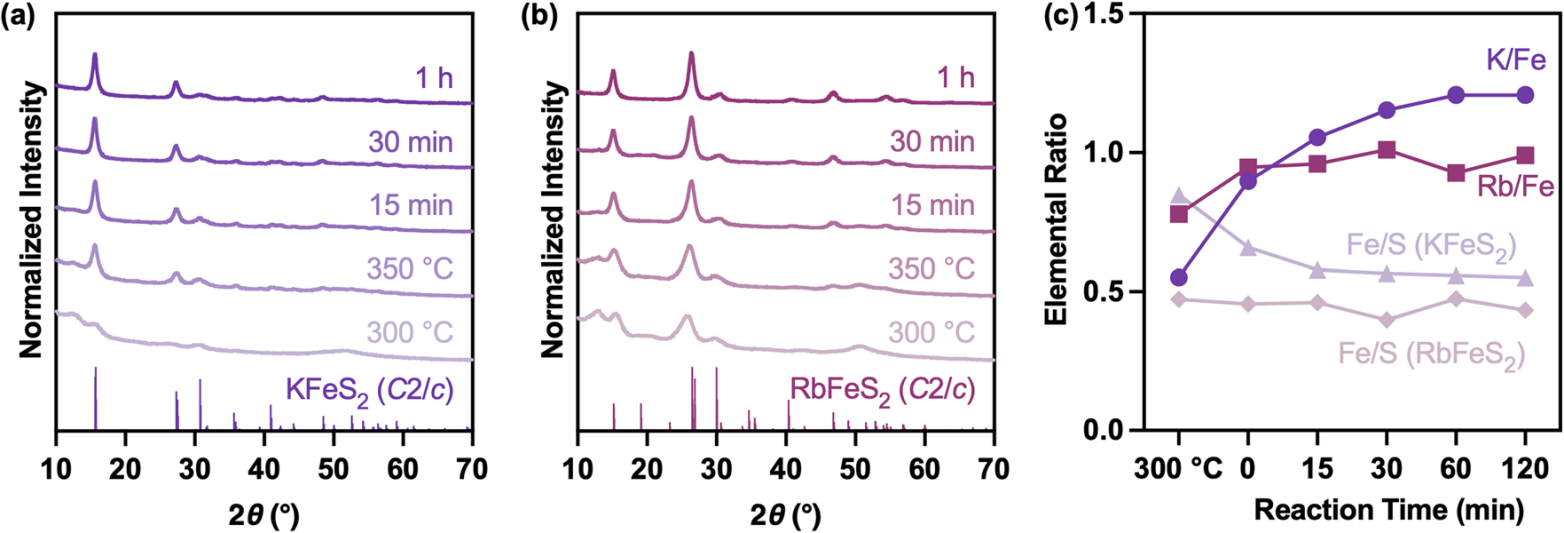
(a and b) Powder XRD patterns and (c) SEM-EDX elemental ratios of aliquots collected during the synthesis of KFeS_2_ and RbFeS_2_ nanowires.

To gain information about the nanowire formation, reaction aliquots were extracted at various temperatures and time intervals. For both KFeS_2_ and RbFeS_2_, powder XRD patterns show the direct nucleation of the monoclinic phase (Fig. 3a and b) by 300 °C, with an extra reflection at 12° 2*θ* that could be ascribed to unreacted precursors. That reflection disappeared within 15 min at 350 °C, forming pure KFeS_2_ and RbFeS_2_ phases. From SEM-EDX analysis (Fig. 3c), RbFeS_2_ established a stoichiometry of Fe : S ≈ 1 : 2 by 300 °C, corresponding to the formation of [FeS_2_]^−^ chains. In contrast, KFeS_2_ started from a sulfur-deficient composition, which plateaued at Fe : S ≈ 1 : 2 after 15 min at 350 °C. Both KFeS_2_ and RbFeS_2_ exhibited a gradual incorporation of alkali metals. After reaching saturation at 350 °C, the Rb^+^ stoichiometry in RbFeS_2_ remained constant throughout the reaction, while K^+^ in KFeS_2_ kept incorporating until it was supersaturated by 20%.

At 300 °C, RbFeS_2_ exhibited both higher crystallinity and a higher alkali metal:Fe ratio than KFeS_2_, indicating a higher reactivity of Rb^+^ than K^+^. This was consistent with our previous observation in the AInSe_2_ (A = K, Rb, Cs) system and can be explained by the hard–soft acid–base (HSAB) theory;^31^ that is, S^2−^ is a relatively soft anion with absolute hardness *η* = 3.87, while Rb^+^ (*η* = 11.55) is softer than K^+^ (*η* = 13.64) and thus shows higher affinity in reacting with the soft S^2−^ anion.^37^

Due to the high surface-to-volume ratio of the ultrathin nanowires, it is essential to evaluate their chemical stability upon heating or air exposure. Stability tests were conducted on nanowire powder samples (Fig. S4). Upon heating at 500 °C and 800 °C for 1 h under flowing N_2_, KFeS_2_ nanowires exhibited only minor decomposition, while RbFeS_2_ nanowires showed no detectable impurity phases. In both cases, sharpening of XRD reflections indicated crystal domain growth, and the emergence of previously suppressed reflections suggests a reduction in preferred orientation. This can be attributed to nanowire fusion following the volatilization of surface ligands, as supported by thermogravimetric analysis (TGA, Fig. S5). After 10 days of air exposure on the benchtop, KFeS_2_ nanowires showed slight degradation, whereas RbFeS_2_ nanowires remained structurally unchanged (Fig. S4). The decomposition products could not be identified, as their XRD patterns do not match any known database entries. Overall, both KFeS_2_ and RbFeS_2_ nanowires exhibit good structural stability under heat and air exposure. For the reliability of results, all other data presented in this work were collected from samples stored under N_2_, with minimal air exposure during characterization.

To evaluate whether the soft-chemistry approach could be extended to other related 1D structures, we explored the synthesis of isostructural KSbS_2_ (Fig. S6a). Using antimony(iii) acetate as the Sb precursor, near-stoichiometric KSbS_2_ was obtained after a 15 min reaction at 350 °C (Fig. S6b and Table S2). However, SEM imaging revealed the formation of large, isotropic nanocrystals with diameters of several hundred nm (Fig. S6c), which was also corroborated by the narrow XRD peak widths indicative of large crystallites. An aliquot study revealed that the reaction proceeded *via* a cubic Sb_2_O_3_ (*Fd*3̄*m*) intermediate, which was already present in the 150 °C sample collected immediately after the degassing step (Fig. S6b). The intermediate Sb_2_O_3_ phase persisted up to 250–300 °C, followed by a quick transition to KSbS_2_ by 350 °C. Notably, the morphology and size of the Sb_2_O_3_ particles observed at 250 °C closely resembled those of the final KSbS_2_ product, suggesting a template-like role during phase evolution (Fig. S6d). Isotropic Sb_2_O_3_ crystals of similar sizes have been previously synthesized by the hydrolysis of SbCl_3_ in oleylamine/oleic acid, the same solvent system used in this study.^38^ This Sb_2_O_3_ intermediate appears to inhibit the formation of the desired ultrathin nanowire morphology by dominating isotropic nucleation and rapid growth. It reveals that the colloidal synthesis of non-vdW 1D materials is system-dependent, requiring not only structural anisotropy in the target phase but also carefully tuned precursor reactivities to enable direct nucleation of the anisotropic product. In the case of KSbS_2_, the formation of Sb_2_O_3_ appears to be kinetically rather than thermodynamically driven. Although Sb^3+^ is known to be sulfophilic rather than oxophilic—given its major mineral form, stibnite (Sb_2_S_3_)—Sb_2_O_3_ nanocrystals nucleated at relatively low temperatures (<150 °C) in this reaction, before the activation of S^2−^ and K^+^.^39^ It may require the use of oxygen-free metal precursors, solvents, and highly reactive sulfur sources to promote direct conversion to the desired KSbS_2_ phase.

**Fig. 4:**
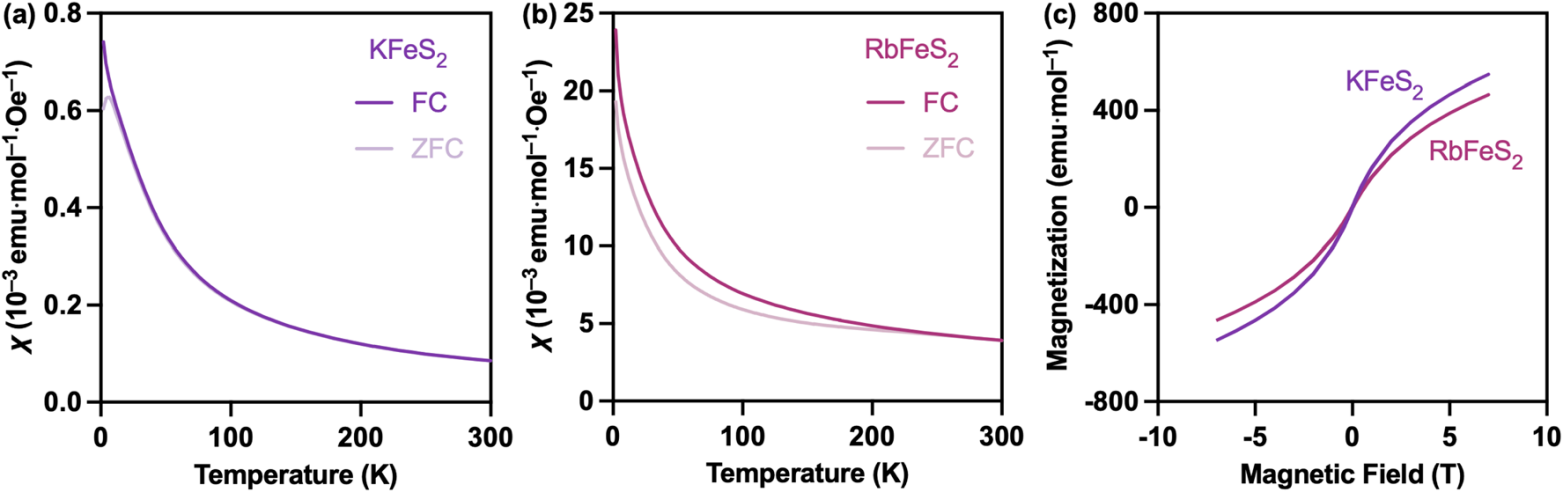
Magnetic characterizations of KFeS_2_ and RbFeS_2_ nanowires. (a and b) Temperature-dependent zero-field-cooled (ZFC) and field-cooled (FC) magnetic susceptibility (*χ*). (c) Field-dependent magnetization at 5 K.

Finally, we studied the magnetic behavior of the ultrathin KFeS_2_ and RbFeS_2_ nanowires, which exhibit significantly lower AFM transition temperatures and completely suppressed interchain FM interactions. Sample masses were calibrated based on TGA to account for surface ligands (Fig. S5). Fig. 4a and b show the temperature-dependent magnetic susceptibility data collected under zero-field-cooled (ZFC) and field-cooled (FC) conditions for KFeS_2_ and RbFeS_2_ nanowires. For KFeS_2_, the ZFC curve revealed a distinct AFM transition around 6 K; for RbFeS_2_, no clear transition was observed down to 2 K. These transition temperatures are significantly lower than those reported for bulk KFeS_2_ (250 K), bulk RbFeS_2_ (188 K), and chemically exfoliated KFeS_2_ nanomaterials (85 K), highlighting the strong influence of dimensional confinement.^23,24,40^ For RbFeS_2_, a divergence between the ZFC and FC curves at approximately 200 K suggests the presence of magnetic correlations. A Curie–Weiss fit was applied to the high-temperature range of 200–300 K (Fig. S7a). The calculated effective magnetic moment *μ*_eff_ = 4.56 μ_B_, was lower than the expected spin-only value of 5.92 μ_B_ for a high-spin Fe^3+^ ion (3d^5^), implying the persistence of paramagnetic behavior above 300 K. This is further supported by the absence of a plateau in the *χT vs. T* plot up to 300 K (Fig. S7b), suggesting persistent magnetic correlations or short-range interactions well above the room-temperature regime. The negative Weiss constant (*θ* = −53 K) obtained from the fit indicates predominantly antiferromagnetic interactions. It is important to note that due to the presence of various ligands in both samples, diamagnetic corrections could not be applied.

Spin dimer analyses were conducted to estimate the relative strengths of various magnetic exchange interactions,^41^ with various *J* values listed in Table S4. In both structures, the intrachain interactions—those between edge-sharing tetrahedra along the crystallographic *c*-axis—were found to be dominant. However, interchain interactions, although weaker, were not negligible and contributed to the overall magnetic behavior. Indeed, bulk KFeS_2_ has been reported to exhibit both strong intrachain AFM interactions and weak but detectable interchain FM interactions.^24^ Chemically exfoliated KFeS_2_ nanomaterials showed a further increase in hysteresis, which was ascribed to degradation and interchain collapse (reduced Fe–Fe distance) during exfoliation.^24^ In contrast, field-dependent magnetization measurements on our colloidal KFeS_2_ and RbFeS_2_ nanowires revealed zero hysteresis at 5 K (Fig. 4c), indicating the absence of even soft FM behavior. This suggests that interchain FM interactions are effectively suppressed in the ultrathin nanowire morphology, likely due to increased chain separation and reduced interchain coupling. The lack of FM signatures also points to the structural integrity of the colloidally synthesized nanowires, with no evidence of magnetic impurities or defect-induced FM contributions.

## Conclusions

In conclusion, we successfully utilized a soft-chemistry method to synthesize ultrathin KFeS_2_ and RbFeS_2_ nanowires, which are a few nm in width and several μm in length. The nanowires grow preferentially along the *c*-axis, parallel to the covalent [FeS_2_]^−^ chains in the monoclinic crystal structure. Aliquot studies revealed that direct nucleation of the target phases enables the evolution of highly anisotropic morphologies. In contrast, synthesis of the isostructural KSbS_2_ yielded large, isotropic nanocrystals due to a formation pathway *via* an Sb_2_O_3_ intermediate. The ultrathin KFeS_2_ and RbFeS_2_ nanowires exhibit pronounced size and morphology-dependent magnetic behavior. Their antiferromagnetic transition temperatures are significantly lower compared to their bulk and chemically exfoliated counterparts. The absence of magnetic hysteresis indicates suppressed interchain ferromagnetic interactions, attributed to the thin cross-section and structural integrity of the nanowires. These findings demonstrate that soft-chemistry approaches offer a viable route to non-van der Waals 1D nanomaterials, expanding synthetic access to highly anisotropic low-dimensional systems. Future efforts should focus on extending this strategy to other non-van der Waals 1D and 2D structures, and on refining precursor design to eliminate isotropic intermediates in systems like KSbS_2_.

## Experimental section

### General considerations

Potassium carbonate (K_2_CO_3_, 99%, Alfa Aesar), rubidium carbonate (Rb_2_CO_3_, 99.8%, Sigma-Aldrich), iron(iii) acetylacetonate (Fe(acac)_3_, 97%, Sigma-Aldrich), antimony(iii) acetate (Sb(OAc)_3_, 99.99%, Sigma-Aldrich), dibenzyl disulfide (Bn_2_S_2_, 98+%, Alfa Aesar), oleylamine (70%, Sigma-Aldrich), and oleic acid (90%, Sigma-Aldrich) were obtained as indicated. Oleylamine and oleic acid were degassed under vacuum at 120 °C for 4 h before use. Reactions were conducted under a nitrogen atmosphere by using standard Schlenk techniques. All reactions employed J-KEM temperature controllers with *in situ* thermocouples to control and monitor the temperature of the reaction vessel.

### Synthesis of KFeS_2_ and RbFeS_2_ nanowires

K_2_CO_3_ (0.800 mmol, 0.110 g) or Rb_2_CO_3_ (0.800 mmol, 0.185 g), Fe(acac)_3_ (0.800 mmol, 0.282 g), and Bn_2_S_2_ (1.60 mmol, 0.394 g) were placed in a three-neck round-bottom flask and dissolved in 18 mL of oleylamine and 2 mL of oleic acid. The flask was then heated to 140 °C and degassed for 1 h under vacuum. At this point, the solution exhibited a black color, and no more gas evolution was observed, indicating the full transformation of alkali metal carbonates into oleates. The reaction temperature was then ramped to 350 °C under flowing nitrogen at 15 °C min^−1^ and held at that temperature for 2 h to yield a black suspension. The reaction suspension was then thermally quenched in a room-temperature water bath. Hexanes (10 mL) were added to the reaction suspension, which was then removed from the round-bottom flask and split equally between three 50 mL centrifuge tubes that were filled to 45 mL with ethanol, sonicated for 3 min, and centrifuged at 6000 rpm for 5 min. This washing procedure was repeated twice, with 7.5 mL of hexanes used to redisperse the nanowires and 37.5 mL of ethanol as the antisolvent. Finally, the precipitates were combined and dispersed in 10 mL of hexanes to form a flocculent suspension, which could be subsequently dried to a powder for characterization. Typically, >0.700 mmol of KFeS_2_ and RbFeS_2_ nanowires can be recovered from each reaction (with ligand mass calibrated by TGA), corresponding to a yield of >87%. Minor losses were observed during purification due to the loose packing of nanowire pellets. For the preparation of TEM samples, the suspensions were diluted 100 times in hexanes, and appropriate amounts of oleylamine were added to reduce agglomeration.

### Aliquot studies

At designated temperature or reaction time, 2 mL fractions of reaction suspensions were extracted from the round-bottom flask using a glass syringe and swiftly injected into 10 mL of room-temperature ethanol to quench. The syringe was then purged with fresh hexanes at least three times to prevent cross-contamination among aliquots.

### Characterization

Powder X-ray diffraction (XRD) measurements were collected on a Rigaku Miniflex powder X-ray diffractometer using Cu Kα radiation (*λ* = 1.541 Å). Powder samples were prepared on a zero-diffraction silicon substrate. Rietveld structural refinements were performed using the BGMN/Profex 5.2.0 software.^42,43^ Refined parameters included scale factors, background, peak shapes, crystallite size/microstrain broadening, lattice constants, atomic positions (if symmetry-allowed), preferred orientation effects, and isotropic thermal parameters. Structure .cif files for KFeS_2_ (ICSD-68383) and RbFeS_2_ (ICSD-202381) from the Inorganic Crystal Structure Database (ICSD) were used for the refinements.

High resolution X-ray photoelectron spectroscopy (XPS) was performed on powdered samples supported on Cu plates, using an ESCALAB QXi system equipped with a monochromated, micro-focused Al Kα X-ray source, a bi-polar hemispherical analyzer, and a flood gun for charge compensation. Binding energies were referenced to the C 1s core level at 284.0 eV.

Scanning electron microscopy (SEM) and energy dispersive X-ray spectroscopy (SEM-EDX) were performed on powder samples supported on copper plates, using an Apreo 2 microscope at an operating voltage of 30 kV, equipped with an Oxford UltimMax 170 silicon drift EDX detector. Transmission electron microscopy (TEM) was performed on dropcast samples supported on lacey carbon-coated copper TEM grids (Ted Pella, Inc.). The prepared grids were placed in a vacuum oven overnight for the removal of volatile organics. TEM imaging was performed on an FEI Talos F200C G2 microscope at an operating voltage of 200 kV, equipped with a Ceta complementary metal-oxide-semiconductor (CMOS) camera. Fast Fourier Transform (FFT) filtering was performed using the ImageJ software by selectively masking visible diffraction spots in FFT patterns, followed by an inverse FFT to obtain the filtered images. EDX quantification was performed using the Aztec software from Oxford Instruments. Emission lines used for quantification are: K series for S, K, Fe, and Rb, and L series for Sb.

Thermogravimetric analysis (TGA) was performed on a TA Instruments TGA Q50 instrument, and samples were analyzed in an alumina crucible under a flowing nitrogen atmosphere. The actual mass of the sample for magnetic measurements was calibrated using the mass percentage obtained from TGA (Fig. S5).

Temperature-dependent direct current (DC) magnetic susceptibility and isothermal (at 5 K) field-dependent magnetization were measured using a Quantum Design SQUID MPMS-3 magnetometer. Powder samples were loaded into gel capsules and mounted in plastic straws for measurements. Both zero-field-cooled (ZFC) and field-cooled (FC) data were collected over the temperature range of 2 K to 300 K under an applied magnetic field of 1000 Oe.

## Author contributions

Z. Sun: data curation, formal analysis, investigation, writing – original draft; N. Pham: data curation, formal analysis, writing – review & editing; S. Derakhshan: funding acquisition, project administration, supervision, writing – review & editing; R. L. Brutchey: conceptualization, funding acquisition, project administration, supervision, writing – review & editing.

## Conflicts of interest

The authors declare no competing financial interest.

## Supplementary Material

SC-016-D5SC04592D-s001

## Data Availability

The data supporting this article have been included as part of the SI. Supplementary information is available. See DOI: https://doi.org/10.1039/d5sc04592d.
