# Supplementary material for: Colloidal synthesis of ultrathin KFeS2 and RbFeS2 magnetic nanowires with non-van der Waals 1D structures
Source: Chem Sci. 2025 Sep 12;16(40):18722–8. doi: 10.1039/d5sc04592d (PMC12428650; doi:10.1039/d5sc04592d)
Supplement: SC-016-D5SC04592D-s001 [file SC-016-D5SC04592D-s001.pdf]

Supplementary Information (SI) for Chemical Science.
This journal is © The Royal Society of Chemistry 2025

Electronic Supplementary Information

Colloidal synthesis of ultrathin $KFeS_2$ and $RbFeS_2$ magnetic nanowires with non-van der Waals 1D structures

Zhaohong Sun,[a] Ngoc Pham,[b] Shahab Derakhshan,*[b] and Richard L. Brutchey*[a]

[a] Department of Chemistry, University of Southern California, Los Angeles, California 90089, United States. Email: brutchey@usc.edu
[b] Department of Chemistry and Biochemistry, California State University, Long Beach, California 90840, United States. Email: shahab.derakhshan@csulb.edu

Additional experimental details

Synthesis of $KSbS_2$ nanocrystals. K_2CO_3 (0.800 mmol, 0.110 g), $Sb(OAc)_3$ (0.800 mmol, 0.239 g), and Bn_2S_2 (1.60 mmol, 0.394 g) were placed in a three-neck round-bottom flask and dissolved in 18 mL of oleylamine and 2 mL of oleic acid. The flask was then heated to 140 °C and degassed for 1 h under vacuum. At this point, the solution had a cloudy orange color, and no more gas evolution was observed, indicating the full transformation of K_2CO_3 into K(oleate). The reaction temperature was then ramped to 350 °C under flowing nitrogen at 15 °C/min and held at that temperature for 15 min to yield a dark yellow suspension. The reaction suspension was then thermally quenched by placing it in a room-temperature water bath. Hexanes (10 mL) were added to the reaction suspension, which was then removed from the round-bottom flask and split equally between three 50 mL centrifuge tubes that were filled to 45 mL with ethanol, sonicated for 3 min, and centrifuged at 6000 rpm for 5 min. This washing procedure was repeated twice, with 7.5 mL of hexanes used to redisperse the nanocrystals and 37.5 mL of ethanol as the antisolvent. The nanocrystal precipitates were dispersed in hexanes or dried to a powder for characterization.

Spin dimer analyses. Spin dimer analyses, based on extended Hückel calculations, were performed to evaluate the relative strengths of various magnetic exchange interactions. Inter-site hopping energy values $\langle \Delta e \rangle$ were computed for different dimers, namely $[Fe_2S_6]^{6-}$, representing intrachain interactions, and $[Fe_2S_8]^{10-}$, for inter-chain interactions, using the CAESAR[1] and SAMOA[#] computational packages. For these calculations, double-zeta Slater-type orbitals (STOs) were employed for sulfur 3*s* and 3*p* and iron 3*d* orbitals, while single-zeta STOs were used for the iron 4*s* and 4*p* orbitals. The parameters used in the extended Hückel approach—including the Slater orbital exponents (ζ_i and ζ'_i) and valence shell ionization potentials, H_{ii}, are provided in **Table S3**. The exchange interaction in each pathway is related to its corresponding hopping energy by:

$$J \propto \frac{\langle (\Delta e)^2 \rangle}{U} \qquad \text{(S1)}$$

where J is the exchange interaction strength and U represents the on-site Coulomb repulsion energy, thus stronger hopping between magnetic centers leads to enhanced magnetic exchange interactions. The U values are identical for all pathways. As a result, the relative magnitudes of

the exchange interactions (J) can be estimated by comparing the corresponding $\langle \Delta e \rangle^2$ values. Since both e and t_2 states (in tetrahedral crystal field) contribute to magnetic interactions along the exchange pathways, the $\langle \Delta e \rangle^2$ values were calculated using the following equation:

$$\langle (\Delta e)^2 \rangle = \frac{1}{25} [(\Delta e_{11})^2 + (\Delta e_{22})^2 + (\Delta e_{33})^2 + (\Delta e_{44})^2 + (\Delta e_{55})^2] \quad \text{(S2)}$$

where Δe_{ii} is the individual hopping integrals between magnetic orbitals involved in the exchange pathway. This summation includes contributions from all relevant orbital overlaps influencing the spin exchange.

Four major interactions were considered: the intra-chain interactions between the edge-sharing tetrahedra (J_1), inter-chain interactions between two separated tetrahedra, in the *ac* crystallographic plane (J_3), and inter-chain interactions between two adjacent *ac* layers, along the *b* direction (J_2 and J_4). The calculated $\langle \Delta e \rangle^2$ values and the relative strengths of J values for both compounds are summarized in **Table S4**.

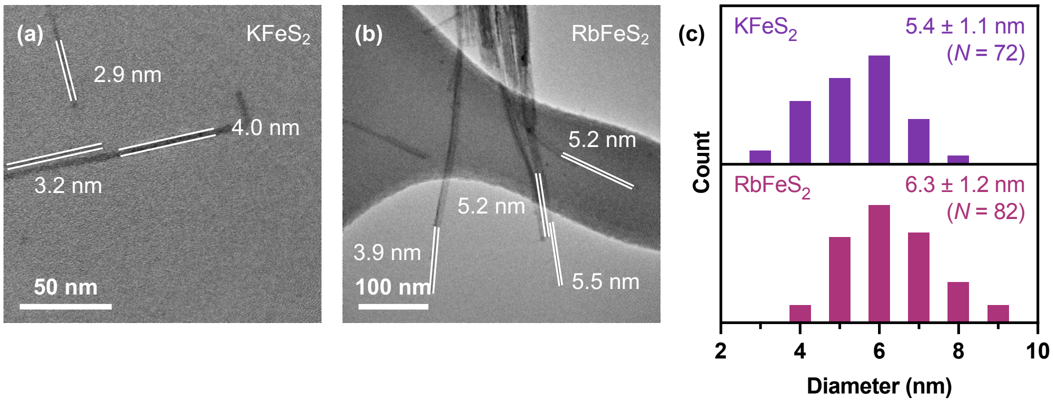

Fig. S1 (a,b) TEM images showing additional diameter measurements of (a) $KFeS_2$ and (b) $RbFeS_2$ nanowires. (c) Nanowire diameter distributions of $KFeS_2$ and $RbFeS_2$.

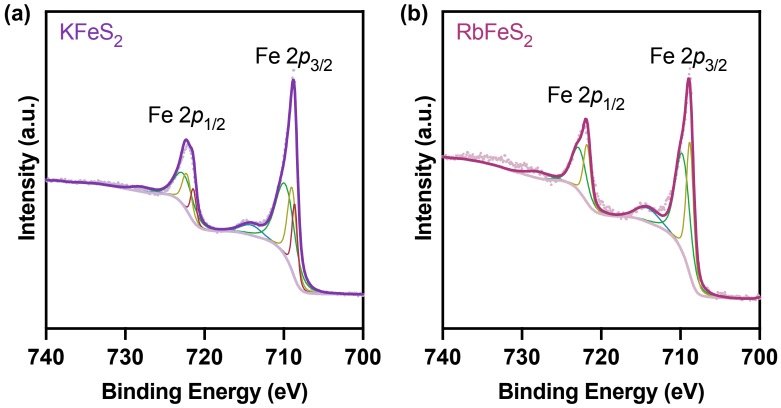

Fig. S2 High-resolution XPS spectra of (a) $KFeS_2$ and (b) $RbFeS_2$ nanowires, revealing the binding energies of Fe 2*p* orbitals.

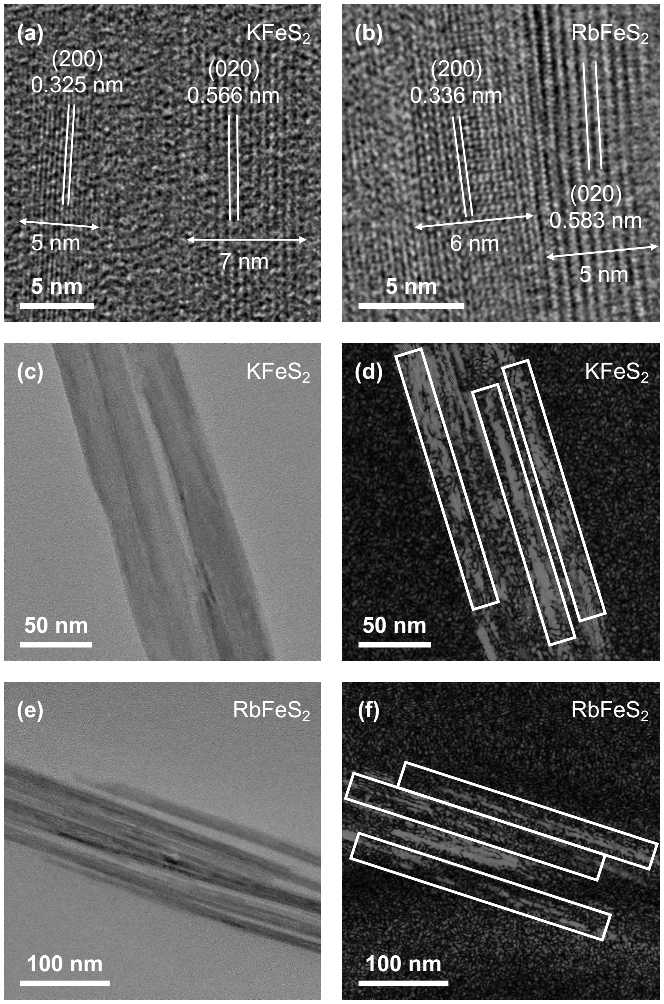

Fig. S3 (a,b) FFT-unfiltered HR-TEM images of (a) $KFeS_2$ and (b) $RbFeS_2$ nanowires, corresponding to the white boxed regions in Fig. 2a,c. (c–f) FFT-unfiltered and filtered images of (c,d) $KFeS_2$ and (e,f) $RbFeS_2$ nanowire bundles, demonstrating the long-range crystallinity of the nanowires. The bright contrast in (d) and (f) results from constructive interference between lattice fringes and FFT filters at the spatial frequencies of the (200) or (020) planes. In rare cases where a nanowire lies flat and is fully in focus at the eucentric height, the *ac* and/or *bc* planes are clearly visible over hundreds of nanometers along the *c*-axis (marked by white boxes). Given the nanowires' flexibility and ultrathin dimension, the absence of lattice fringes in other regions is not indicative of structural disorder, but rather results from slight bending of the nanowires out of the imaging plane.

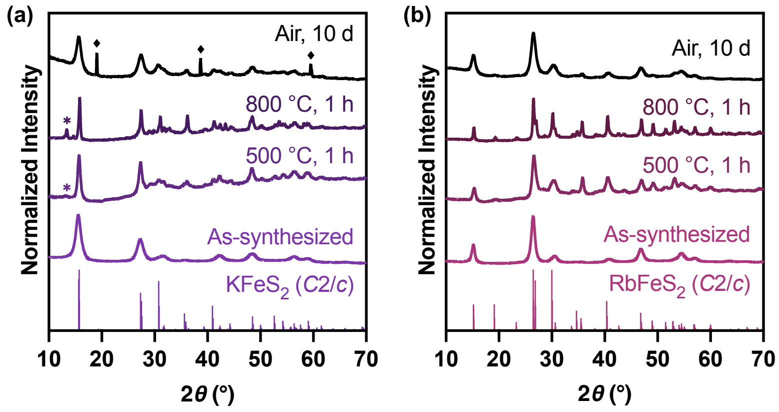

Fig. S4 Powder XRD patterns of (a) $KFeS_2$ and (b) $RbFeS_2$ nanowires upon heating under N_2 or exposure to air. In the case of $KFeS_2$, additional reflections attributed to decomposition products are marked with "✱" and "♦". The exact identities of these phases have not yet been determined.

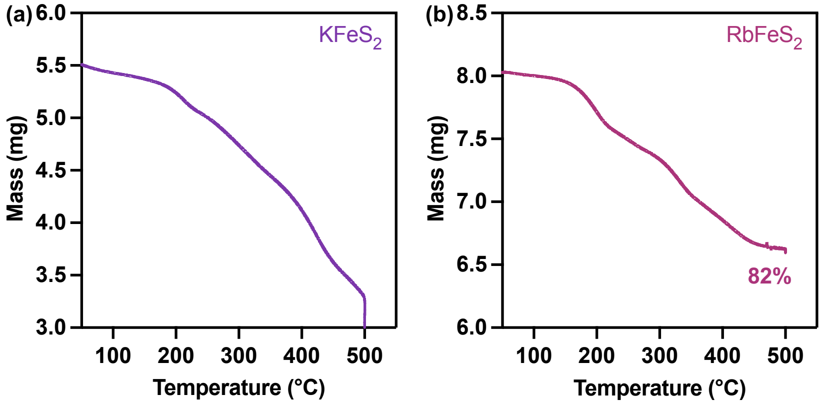

Fig. S5 TGA curves of (a) $KFeS_2$ and (b) $RbFeS_2$ nanowires. After ligand volatilization, $RbFeS_2$ retained 82% of its original mass at 500 °C, which was used to calibrate sample mass in magnetic measurements. In contrast, $KFeS_2$ exhibited continuous mass loss during isotherm at 500 °C, indicating thermal decomposition that prevents an accurate mass calibration. Due to the structural and dimensional similarity between the two materials, the 82% residual mass value from $RbFeS_2$ was also applied for the calibration of $KFeS_2$.

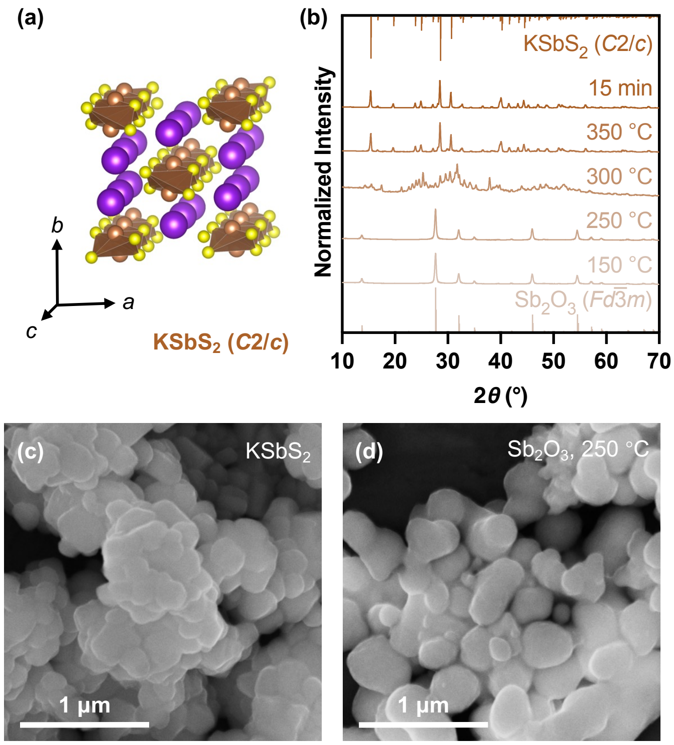

Fig. S6 Formation pathway of $KSbS_2$ nanocrystals. (a) Crystal structure of monoclinic $KSbS_2$ (space group *C*2/*c*), showing $[SbS_2]^-$ chains along the *c*-axis, hexagonally packed among K^+. The $5s^2$ lone pair on Sb^{3+} results in a seesaw coordination geometry rather than tetrahedral, giving the $[SbS_2]^-$ chains a "flatter" appearance compared to their Fe-based counterparts. (b) Powder XRD patterns of aliquots collected during the synthesis of $KSbS_2$ nanocrystals, with reference patterns of cubic Sb_2O_3 (ICSD-1944) and monoclinic $KSbS_2$ (ICSD-60138) included. (c) SEM image of $KSbS_2$ nanocrystals. (d) SEM image of Sb_2O_3 nanocrystals obtained in the 250 °C aliquot.

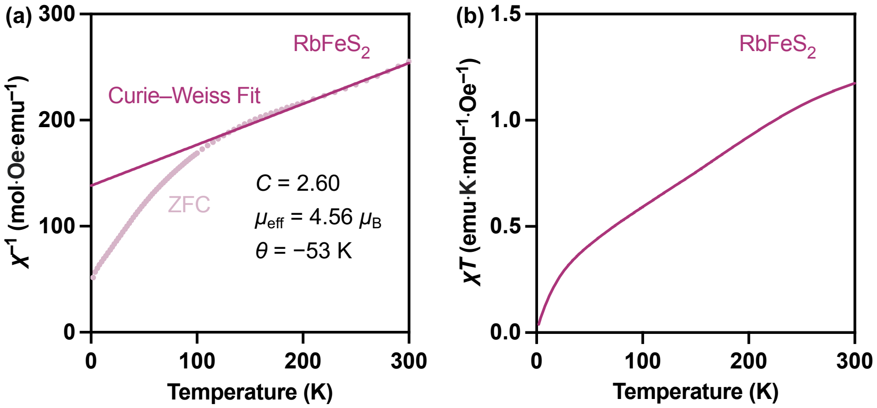

Fig. S7 (a) Curie–Weiss fit of the magnetic susceptibility data for $RbFeS_2$ nanowires in the high-temperature range (200–300 K), along with the extracted fitting parameters. (b) Temperature dependence of χT, revealing that the paramagnetic behavior characterized by a temperature-independent regime in χT (i.e., constant C), is not established for $RbFeS_2$ nanowires, even at room temperature.

Table S1. Refined lattice parameters of $KFeS_2$ and $RbFeS_2$ nanowires compared to literature values of bulk single crystals.[2]

	$KFeS_2$		$RbFeS_2$	
	Refinement	Literature	Refinement	Literature
a (Å)	7.028(4)	7.084(3)	7.241(2)	7.223(3)
b (Å)	11.317(3)	11.303(4)	11.665(2)	11.725(3)
c (Å)	5.424(6)	5.394(2)	5.452(5)	5.430(2)
β (°)	112.73(8)	113.2(1)	112.07(4)	112.0(1)
V (Å^3)	397.9	397.0	426.8	426.4
K/Rb in 4e				
x	0.0	0.0	0.0	0.0
y	0.3617(2)	0.3572(1)	0.3548(2)	0.3553(2)
z	0.25	0.25	0.25	0.25
Fe in 4e				
x	0.0	0.0	0.0	0.0
y	0.9980(3)	0.99668(8)	1.0058(7)	0.9976(3)
z	0.25	0.25	0.25	0.25
S in 8f				
x	0.1825(6)	0.1960(2)	0.160(1)	0.1905(6)
y	0.1045(2)	0.1098(1)	0.1036(2)	0.1061(4)
z	0.1068[a]	0.1068(2)	0.0981[a]	0.0981(7)

[a] Due to the strong preferred orientation of nanowires, the z coordinates of S^{2-} were fixed at literature values to prevent overfitting.

Table S2. Elemental compositions of samples acquired by SEM-EDX. All atomic percentages exceeded three times their respective errors, ensuring statistical significance.

Aliquot study, $KFeS_2$					
Aliquot	K at%	Fe at%	S at%	K/Fe	S/Fe
300 °C	20.20(11)	36.63(10)	43.17(16)	0.55	1.18
350 °C, 0 min	26.27(15)	29.28(12)	44.45(19)	0.90	1.52
15 min	27.88(9)	26.42(7)	45.69(11)	1.06	1.73
30 min	29.41(8)	25.50(6)	45.09(10)	1.15	1.77
1 h	30.19(9)	25.00(6)	44.81(11)	1.21	1.79
2 h	30.03(7)	24.87(6)	45.10(9)	1.21	1.81
Aliquot study, $RbFeS_2$					
Aliquot	Rb at%	Fe at%	S at%	K/Fe	S/Fe

300 °C	19.99(27)	25.69(23)	54.33(47)	0.78	2.11
350 °C, 0 min	22.86(18)	24.14(15)	53.00(31)	0.95	2.20
15 min	23.24(12)	24.21(10)	52.56(22)	0.96	2.17
30 min	22.37(18)	22.14(15)	55.49(34)	1.01	2.51
1 h	22.98(11)	24.79(10)	52.23(20)	0.93	2.11
2 h	23.03(12)	23.26(11)	53.71(22)	0.99	2.31
$KSbS_2$					
Sample	K at%	Sb at%	S at%	K/Sb	S/Sb
$KSbS_2$	25.30(32)	25.89(15)	48.80(43)	0.98	1.88

Table S3. Slater orbital exponents (ζ_i and ζ'_i) and valence shell ionization potentials (H_{ii}) of the atomic STO's employed for the spin dimer calculations for $KFeS_2$ and $RbFeS_2$.

Atom	Orbital	H_{ii} (eV)	ζ_i	C	ζ'_i	C'
S	3*s*	–20.00	2.662	0.5564	1.688	0.4873
S	3*p*	–13.300	2.338	0.5212	1.333	0.5443
Fe	4*s*	–9.100	1.900	1		
Fe	4*p*	–5.320	1.390	1		
Fe	3*d*	–12.600	5.350	0.5505	2.000	0.6260

Table S4. The calculated $\langle \Delta e \rangle^2$ values and the relative strengths of J values for $KFeS_2$ and $RbFeS_2$.

	$KFeS_2$				$RbFeS_2$			
	J_1	J_2	J_3	J_4	J_1	J_2	J_3	J_4
$\langle \Delta e \rangle^2$ (meV^2)	2174	256	310	274	2009	393	421	399
Relative values	1.00	0.12	0.14	0.13	1.00	0.20	0.21	0.20

References

1 J. Ren, W. Liang and M. H. Whangbo, *Crystal and Electronic Structure Analysis Using CAESAR*, 2005, http://www.primeC.com.

2 W. Bronger, A. Kyas and P. Müller, *J. Solid State Chem.*, 1987, **70**, 262–270.

Our calculations employed the SAMOA (Structure and Molecular Orbital Analyzer) program package, which can be downloaded free of charge from: http://chvamw.chem.ncsu.edu/ (accessed Apr 2008).
